# Circadian regulation of hippocampal function is disrupted with corticosteroid treatment

**DOI:** 10.1073/pnas.2211996120

**Published:** 2023-04-06

**Authors:** Matthew T. Birnie, Matthew D. B. Claydon, Oliver Troy, Benjamin P. Flynn, Mitsuhiro Yoshimura, Yvonne M. Kershaw, Zidong Zhao, Rebecca C. R. Demski-Allen, Gareth R. I. Barker, E. Clea Warburton, Zuner A. Bortolotto, Stafford L. Lightman, Becky L. Conway-Campbell

**Affiliations:** ^a^Henry Wellcome Laboratories for Integrative Neuroscience and Endocrinology, Translational Health Sciences, Faculty of Health Sciences, School of Medicine, University of Bristol, Bristol BS1 3NY, United Kingdom; ^b^School of Physiology, Pharmacology and Neuroscience, Faculty of Life Sciences, University of Bristol, Bristol BS8 1TD, United Kingdom

**Keywords:** glucocorticoids, hippocampus, circadian rhythms, memory, methylprednisolone

## Abstract

Disrupted circadian activity is associated with many neuropsychiatric disorders. A major coordinator of circadian biological systems is adrenal glucocorticoid secretion which exhibits a pronounced preawakening peak that regulates metabolic, immune, and cardiovascular processes, as well as mood and cognitive function. Loss of this circadian rhythm during corticosteroid therapy is often associated with memory impairment. Surprisingly, the mechanisms that underlie this deficit are not understood. In this study, in rats, we report that circadian regulation of the hippocampal transcriptome integrates crucial functional networks that link corticosteroid-inducible gene regulation to synaptic plasticity processes via an intrahippocampal circadian transcriptional clock. Further, these circadian hippocampal functions were significantly impacted by corticosteroid treatment delivered in a 5-d oral dosing treatment protocol. Rhythmic expression of the hippocampal transcriptome, as well as the circadian regulation of synaptic plasticity, was misaligned with the natural light/dark circadian-entraining cues, resulting in memory impairment in hippocampal-dependent behavior. These findings provide mechanistic insights into how the transcriptional clock machinery within the hippocampus is influenced by corticosteroid exposure, leading to adverse effects on critical hippocampal functions, as well as identifying a molecular basis for memory deficits in patients treated with long-acting synthetic corticosteroids.

## Significance

Circadian oscillations of glucocorticoids are a fundamental characteristic of adrenal hormone secretion in all mammals. These rhythms can be disrupted by chronic stress or synthetic glucocorticoid therapy, resulting in side effects including impaired memory, mood, and sleep. Here, we show that 5-d treatment with methylprednisolone induces prolonged activation of glucocorticoid receptors and consequent binding at glucocorticoid regulatory elements in the core clock gene *Period 1*, in the hippocampus. Furthermore, disruption of circadian hippocampal gene regulation was evident, along with loss of circadian variation in hippocampal long-term potentiation (LTP) and impaired hippocampal memory. Together, these data demonstrate dysregulated hippocampal function during long-acting glucocorticoid treatment and further support the importance of devising improved regimens of glucocorticoid therapy that recapitulate endogenous glucocorticoid rhythmicity.

Circadian rhythms regulate many physiological, biological, and behavioral processes in mammals (1–4). In addition to the primary mammalian clock in the hypothalamic suprachiasmatic nucleus (SCN) (5), which is largely regulated by the light/dark period, clock gene transcriptional rhythms are observed in other regions of the brain, including the hippocampus (6–8), which remain synchronized to the SCN via a combination of neural and humoral signals (4, 5, 9)

The endogenous corticosteroid hormone (predominantly cortisol in man, and corticosterone in rat) is under circadian control via SCN regulation of hypothalamic–pituitary–adrenal (HPA) axis function. Disruption to this, for example in Cushing’s disease and during commonly prescribed steroid therapy for inflammatory diseases such as rheumatoid arthritis or asthma, results in elevated cortisol or exogenous steroid levels, respectively, throughout the day–and has been associated with depression, anxiety, and psychosis (10–12). Recent studies have demonstrated the importance of normal physiological oscillations of steroid availability for optimal cognitive and emotional responses (13), suggesting that the timing of corticosteroid secretion and therefore activity of glucocorticoid receptors (GRs) at specific times of day may be essential to neuropsychiatric health. How synthetic steroid use causes adverse neuropsychiatric and cognitive outcomes, however, remains unknown. In this study, we sought to mimic oral corticosteroid dosing in a rat model, in order to identify how the treatment may influence such adverse outcomes. Methylprednisolone (MPL), which is a long-acting (14), frequently prescribed synthetic corticosteroid (15) associated with circadian disturbances, psychiatric disorders, and memory impairment in humans, (16–18) was provided for 5 d ad libitum in drinking water at a concentration of 1 mg/mL; a treatment regime that will be referred to throughout the text as “MPL treatment.”

**Fig. 1.**
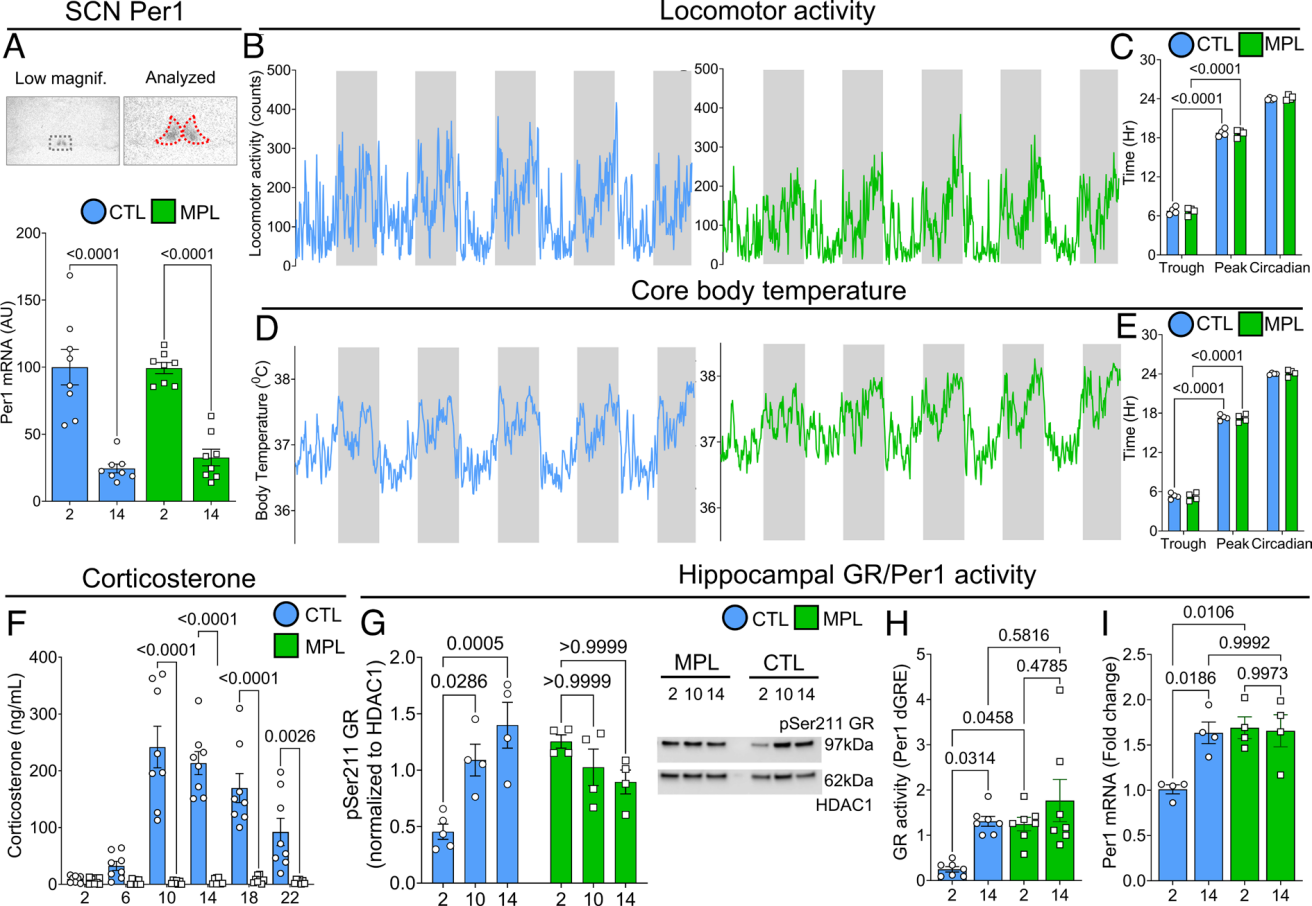
Methylprednisolone treatment desynchronizes hippocampal glucocorticoid receptor activity from endogenous master clock function. (*A*) *Per1* mRNA expression in the SCN in CTL and MPL-treated rats. *F*_1,28_ = 83.97, *P* < 0.0001, for time by two-way ANOVA with Sidak’s post-hoc test. (*B*) Example locomotor activity in a CTL (blue) and a MPL (green)-treated rat. (*C*) CTL and MPL-treated rats maintained rhythmic locomotor activity (*F*_1,12_ = 1801, *P* < 0.0001: CTL; *P* < 0.0001, MPL; *P* < 0.0001). (*D*) Example core body temperature in a CTL (blue) and a MPL (green)-treated rat. Similar to activity data, (*E*) MPL treatment did not influence core body temperature rhythm (*F*_1,12_ = 1,964, *P* < 0.0001: CTL; *P* < 0.0001, MPL; *P* < 0.0001). Two-way ANOVA with Sidak’s post-hoc test. (*F*) MPL in drinking water was sufficient to suppress corticosterone release in rats. *F*_5,81_ = 16.98, *P* < 0.0001, for time, and *F*_1,81_ = 160.6, *P* < 0.0001, for treatment, by two-way ANOVA with Sidak’s post-hoc test. (*G*) Western blot showing pSer211 GR and HDAC1 loading control in hippocampal nuclear extract samples prepared from MPL-treated and CTL rats. Associated graph displaying relative quantification of densitometry data. *F*_2,19_ = 13.53, *P* = 0.0002, interaction by two-way ANOVA with Bonferroni’s post-hoc test. (*H*) Hippocampal GR activity on *Per1* dGRE. *F*_1,24_ = 9.803, *P* = 0.0045, for time, and *F*_1,24_ = 8.339, *P* = 0.0081, for treatment, by two-way ANOVA and Tukey’s post-hoc test. (*I*) Hippocampal Per1 mRNA expression. *F*_1,12_ = 5.593, *P* = 0.0357, for time, and *F*_1,12_ = 7.925, *P* = 0.0156, for treatment, by two-way ANOVA with Tukey’s post-hoc test. Data are mean ± SEM. *P < 0.05.

We first sought to assess the effects of MPL treatment on the rats’ daily activity profiles, behavior known to be under central clock control, according to zeitgeber time (ZT) (*SI Appendix*, Fig. S1*A*). *Period 1* (*Per1*), a light-entrainable regulator of rhythmic function (19), constitutes a key component of the circadian clock machinery and can be targeted by the GR (*Nr3c1*) via a hypersensitive glucocorticoid response element (GRE) (20). *Per1* is highly enriched in the SCN, where we found similarly robust circadian fluctuation in its messenger RNA (mRNA) expression in both control and MPL-treated rats (Fig. 1*A* and *SI Appendix*, Fig. S1 *B* and *C*). This finding indicates that SCN *Per1* is resistant to transcriptional disruption by corticosteroids, presumably because the SCN is devoid of GR expression (21). Consistent with this, SCN-regulated physiological rhythms such as locomotor activity and core body temperature were *unaffected* by MPL treatment (Fig. 1 *B*–*E*), remaining primarily under the control of SCN transcriptional clock oscillations synchronized to the 12-h light/12-h dark cycle.

**Fig. 2.**
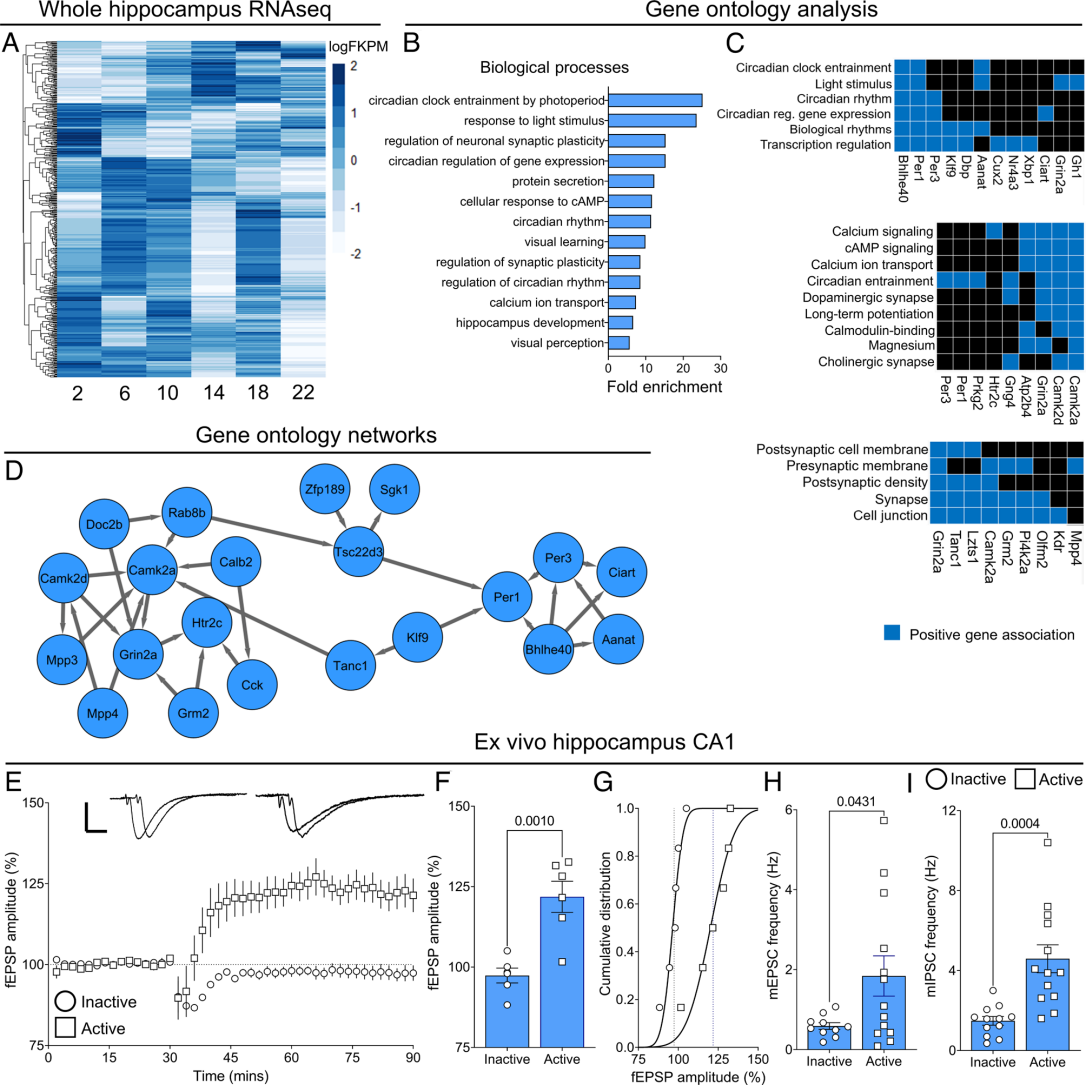
Endogenous regulation of hippocampal function. (*A*) Differentially expressed genes (DEGs; *FDR* < 0.05) across time (*n* = 24, 4/time point). Each row corresponds to a single gene (485 DEGs), each column represents time point. (*B*) Select associated (*P* < 0.05) gene ontology (biological processes) terms among DEGs at ZT 10. (*C*) Functional clustering of DEGs and target pathways at ZT 10. (*D*) Representation of a regulated signaling network enrichment analysis including interacting clock, corticosteroid, and synaptic function DEGs. (*E*–*G*) Evoked long-term potentiation responses from hippocampal CA1 region neurons recorded for 90 min during the early active phase, but not during the early inactive phase (*n* = 5 inactive; *n* = 6 active, *P* = 0.001). (*H* and *I*) mEPSC and mIPSC measured during the early inactive and early active phase (mEPSC *n* = 10 inactive; *n* = 13 active, *P* = 0.0431: mIPSC *n* = 12 inactive; *n* = 13 active, *P* = 0.0004). Data are mean ± SEM. *P < 0.05, by unpaired *t* test (Fig. 2 *F*, *H* and *I*).

In contrast to the SCN, GRs are highly enriched in the hippocampus (21, 22), where their activity displays strong circadian variation in activity (23), in line with the circadian rise and fall in adrenal corticosteroid secretion (Fig. 1*F*), and demonstrated by significantly increased nuclear pSer211 GR during the active (ZT 14) compared to the inactive phase (ZT 2) (Fig. 1*G*), along with significantly increased GR binding to the *Per1* distal GRE (Fig. 1*H*), and subsequent *Per1* transcription (Fig. 1*I*), during the active (ZT 14) but not inactive phase (ZT 2). RNA sequencing (RNAseq) data from rat hippocampi every 4 h (from ZT 2—22) identified 485 differentially expressed genes (DEGs) across time (Fig. 2*A* and Dataset S1). Of these DEGs, 56 genes (10%) were found to be directly regulated by corticosteroids in an independent experiment where adrenalectomized (ADX) rats were infused with an acute infusion of 0.75 mg/mL corticosterone at ZT 2 (*SI Appendix*, Fig. S2 *A*–*C* and Dataset S2).

In nonstress conditions, endogenous corticosterone secretion reaches maximal levels near ZT 10, to modulate the awakening response just prior to the start of the active phase (24), when many other biological processes are initiated (25). Accordingly, it is quite well appreciated that during the early period of the active phase, learning and memory processes are more effective, with performance declining as the day progresses (26–30). Therefore, we used gene ontology (GO) analysis to identify the biological processes and molecular functions that were enriched in the DEG analysis at ZT 10, relative to the circadian nadir ZT 2 when endogenous corticosteroid secretion is lowest (Figs. 1*F* and 2 *B*–*D*). At ZT 10, DEGs included *Per1*, *Per2*, *Per3*, *Bhlhe40*, *CamkII*, *Grin2a,* and *Grin2c*, known mediators of circadian rhythms and entrainment, as well as modulators of hippocampal synaptic function (31). *Per1*, *Grin2a,* and *Grin2b* are examples of genes that were directly regulated by acute corticosteroid treatment in ADX rats (*SI Appendix*, Fig. S2 and Dataset S2), Indeed, GR regulatory sites were identified within or proximal to *Per1*, *Per2*, *Bhlhe40*, *CamkII*, *Grin2a,* and *Grin2b* (32). To prepare for the active period, many biological processes have a priming phase, suggesting that genes controlling synaptic function may be regulated by local clock mechanisms in line with the endogenous corticosteroid peak. We clustered the DEGs at ZT 10, by known association (Fig. 2*C*), discovering functional interactions between core clock gene products—*Per1*, *Per3*, *Dbp*, *Bhlhe40,* and *Aanat*–and the known synaptic function genes *Grin2* and *CaMKII,* suggesting they are poised to mediate clock-coupled behavioral rhythms to influence learning and memory processes (33, 34).

*Grin2a* and *Grin2b* encode for GluN2A and GluN2B, respectively. Endogenous corticosteroids have been reported to rapidly regulate GluN2B-NMDAR membrane trafficking through nongenomic mineralocorticoid (MR) signaling (35). However, corticosteroids bind to GRs in a circadian pattern, whereas MRs are near maximally occupied at both the circadian nadir and peak due to their higher corticosteroid affinity (36). Using a network connectivity map, we identified interactions between enriched genes of corticosteroid-mediated expression, clock gene expression, and synaptic function at ZT 10 (Fig. 2*D*). Identification of genes under circadian and corticosteroid control from independent RNAseq analyses (Fig. 2 and *SI Appendix*, Fig. S2: *Per1, Klf9*, *Klf15*, *Sgk1*, *Zfp189,* and *Tsc22d3)* supports corticosteroid-mediated regulation of clock gene expression (37, 38). Importantly, peak corticosteroid secretion occurs in preparation for increased neurocognitive activity, which, in concert with peak *Per1* clock gene expression in the hippocampus, facilitates action on synaptic plasticity target genes *CamkII* and *Grin2* (Fig. 2*D*).

Indeed, corticosteroid action on memory performance is well established (26, 39). However, seminal studies focusing on the role of corticosteroids have concentrated largely on the response to acute changes in corticosteroids in models of stress (39, 40). Furthermore, although time of day is known to influence memory function (26, 27), different models and tests have identified conflicting outcomes (28–30, 41–43). For example, mice and rats learn maze navigation more effectively during the active phase, yet tone-cued fear conditioning in mice is more readily formed during the inactive phase (41). Moreover, evidence that tasks with a high cognitive demand can themselves serve as zeitgebers suggests that the interaction with time of day is stimulus specific (44). Here, we have identified a molecular basis for the improved performance often observed at the start of the active phase. Given that the colocalization between CaMKII and GluN2B-containing NMDARs at the synapse is critical for long-term potentiation (LTP), a cellular correlate of long-term memory, we hypothesized that this process is under circadian control, and potentially driven by GR-mediated transcriptional rhythms. To test this hypothesis, ex vivo hippocampal slices were prepared from rats culled during either the early active or inactive period to allow time for gene transcription occurring at the respective peak or trough of endogenous corticosteroid secretion to translate to functional proteins at the synapse. Using a high-frequency stimulation protocol, the method used previously to show LTP is modulated by the addition of corticosterone (45), we found that LTP was robustly induced in hippocampal slices prepared from control rats culled during the early active period but not the early inactive period (Fig. 2 *E*–*I*). The circadian regulation of hippocampal synaptic activity was also evident in the increased frequency, but not amplitude, of spontaneous synaptic events (miniature excitatory postsynaptic currents; mEPSCs and miniature inhibitory postsynaptic currents; mIPSCs) recorded from CA1 neurons (Fig. 2 *E*–*I* and *SI Appendix*, Fig. S3 *B* and *C*).

Given the GO pathway predictions for associations between glucocorticoids and the synaptic processes under circadian control here, we next sought to interrogate whether exogenous glucocorticoid treatment would have an impact on these functional outputs of the hippocampus. Indeed, corticosteroid-mediated memory improvement has previously been demonstrated in rats, where an acute corticosterone injection during the circadian nadir enhanced memory consolidation in a hippocampal-dependent task (46). Chronic corticosteroid exposure, on the contrary, has been reported to impair memory in both aversive and nonaversive tasks, and at different times of day (47).

**Fig. 3.**
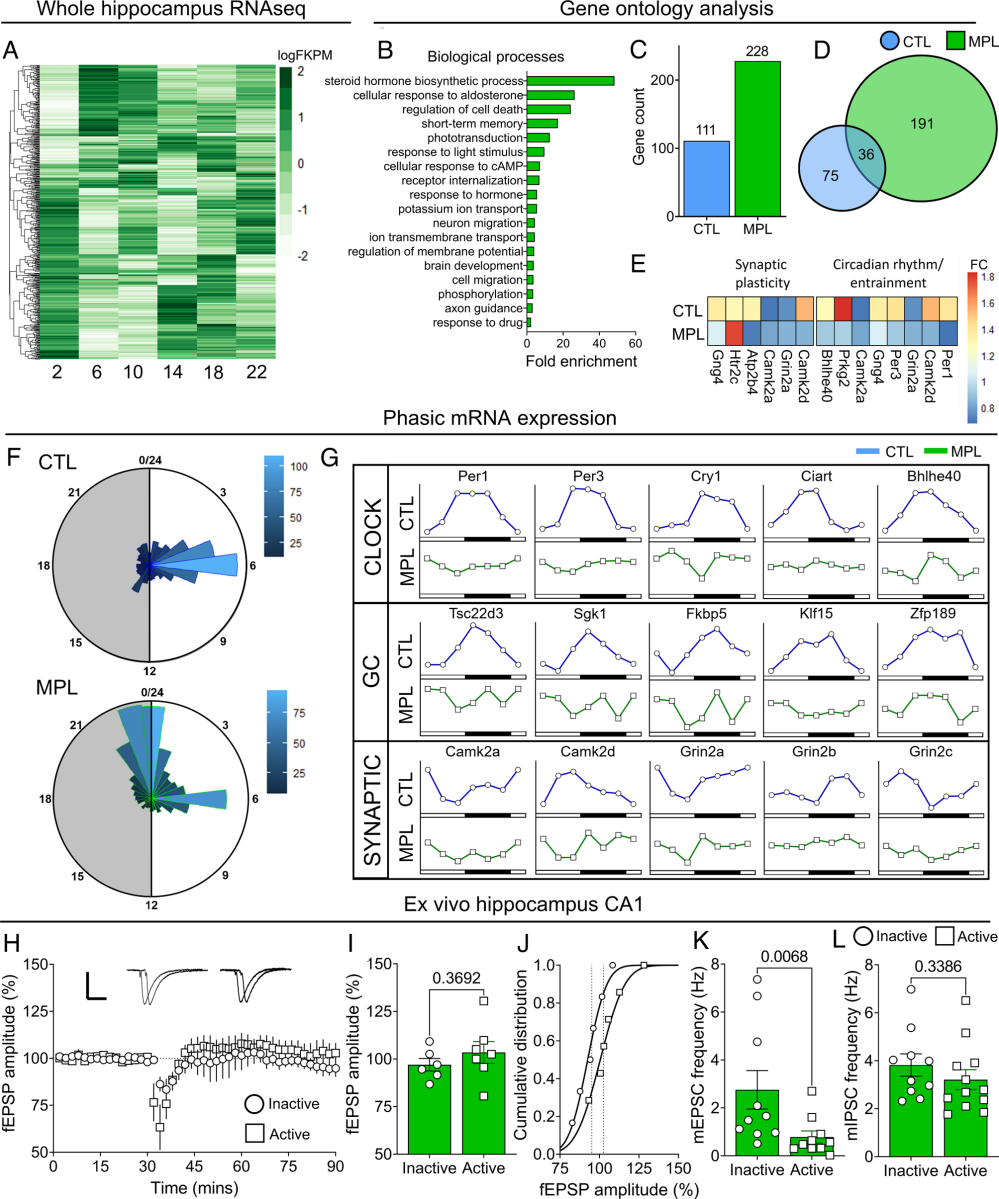
Methylprednisolone disrupts endogenous regulation of hippocampal function. (*A*) Differentially expressed genes (DEGs; *FDR* < 0.05) across time (n = 24, 4/time point). Each row corresponds to a single gene (512 DEGs), each column represents time point. (*B*) Select associated (*P* < 0.05) gene ontology (biological processes) terms of DEGs at ZT 10. (*C*) Total number of DEGs at ZT 10 in CTL and MPL-treated groups. (*D*) Overlap of the DEGs at ZT 10 in CTL and MPL. (*E*) Expression of circadian-regulating and synaptic function genes at ZT 10. (*F*) Distribution of peak expression of DEGs in CTL and MPL groups. (*G*) Gene expression patterns of select clock-regulating, glucocorticoid-target, and synaptic plasticity genes. (*H*–*J*) MPL treatment prevented LTP responses from hippocampal CA1 region neurons during the early inactive and active phases (n = 5 inactive; n = 6 active, *P* = 0.3692), (*K*) mEPSCs recorded during the early inactive and early active phases (n = 10 inactive; n = 10 active, *P* = 0.0068) and (*L*) mIPSCs recorded during the early inactive and early active phases (n = 10 inactive; n = 12 active, *P* = 0.3386). Data are mean ± SEM. *P < 0.05, by unpaired *t* test (Fig. 3 *I* and *K*), or Mann–Whitney *U* test Fig. 3*J*).

We therefore next assessed the effect of 5-d 1 mg/mL MPL treatment (ad libitum in drinking water) on circadian transcriptome regulation and synaptic plasticity in the rat hippocampus. Considering that GR binding dynamics of MPL are distinct from that of endogenous corticosteroid (48), and that MPL treatment protocol suppresses adrenal corticosterone over the full circadian cycle (Fig. 1*F*) while inducing prolonged hippocampal GR activation throughout the full circadian cycle (Fig. 1 *G*–*I*), we hypothesized that MPL treatment may also continuously influence glucocorticoid target genes throughout the day. To test this, we sequenced the RNA from rat hippocampi every 4 h (ZT 2–22), on the final day of MPL-treatment. RNAseq analysis identified 512 DEGs across time (Fig. 3*A* and Dataset S3). Here, we discovered that many genes known to be important for memory processing in the hippocampus were dysregulated with MPL treatment, an important finding considering that circadian disturbances and memory impairments are common among individuals with major depression (49, 50) or that are receiving steroid therapy (51).

Following this, we sought to assess whether the same functional pathways identified in control rat hippocampus were influenced by MPL treatment. GO analysis of DEGs from ZT 10 to ZT 2 comparison (Fig. 3 *B*–*E*) predicted distinct target pathways compared to GO analysis of the equivalent comparison in control data (Fig. 3*B*). It was also notable that additional genes were differentially expressed at ZT 10 in the MPL-treated rats (Fig. 3 *C* and *D* and Dataset S4). Interestingly, no circadian-entraining pathways were identified with GO analysis, suggesting a loss of hippocampal rhythm with MPL treatment. Synaptic plasticity pathways were identified in the MPL treatment ZT 10 vs. ZT 2 comparison, although identified DEGs were again distinct from those identified in the ZT 10 vs. ZT 2 comparison in control rats (Dataset S5). With differential pathway activity between control and MPL-treated groups, we quantified the expression of circadian rhythm and entrainment genes, as well as synaptic plasticity target genes at ZT 10 in control and MPL groups (Fig. 3*E*). Following the detection of a dissociation between clock regulating and synaptic plasticity genes at ZT 10, we sought to determine the peak amplitude of all DEGs across the day (Fig. 3*F* and Dataset S6) (52). We found that most DEGs in control exhibited peak expression levels between ZT 5 and 7 (Fig. 3*F*), whereas following steroid treatment, most DEGs peaked between ZT 22 and 1 (Fig. 3*F*). After identifying a shift in peak gene expression, we sought to identify if this affected the expression patterns of target genes; mapping select expression profiles across time of known clock-regulating, corticosteroid target, and synaptic plasticity-associated genes (Fig. 3*G*). The striking pattern differences, and expression profiles of genes regulating clock and synaptic plasticity processes following MPL treatment, led us to investigate a potential functional output of this disruption.

In controls, LTP was robust during the early active period, as was the frequency of mPSCs (Fig. 2 *E*–*I*). Using the same high frequency stimulation (HFS) protocol, but this time in rats that received 5 d of MPL treatment, LTP could not be induced in either the early active or inactive period (Fig. 3 *H*–*J*). Remarkably, MPL treatment increased the frequency, but not amplitude, of mEPSCs recorded during the early inactive period, an inversion of the pattern observed in controls. This was not evident in the frequency of mIPSC events, instead eliminating circadian variation, suggesting that the MPL treatment disrupted the excitatory/inhibitory (E–I) mPSC balance (Fig. 3 *K* and *L* and *SI Appendix*, Fig. S3 *D* and *E*). Interestingly, alterations to the E–I balance in the forebrain have been implicated in several psychiatric disorders including Alzheimer’s disease (53, 54). Additionally, early-life stress accelerates developmental shifts in the E–I balance (55), emphasizing the role of corticosteroid signaling in potentially mediating the regulation of this phenomenon.

**Fig. 4.**
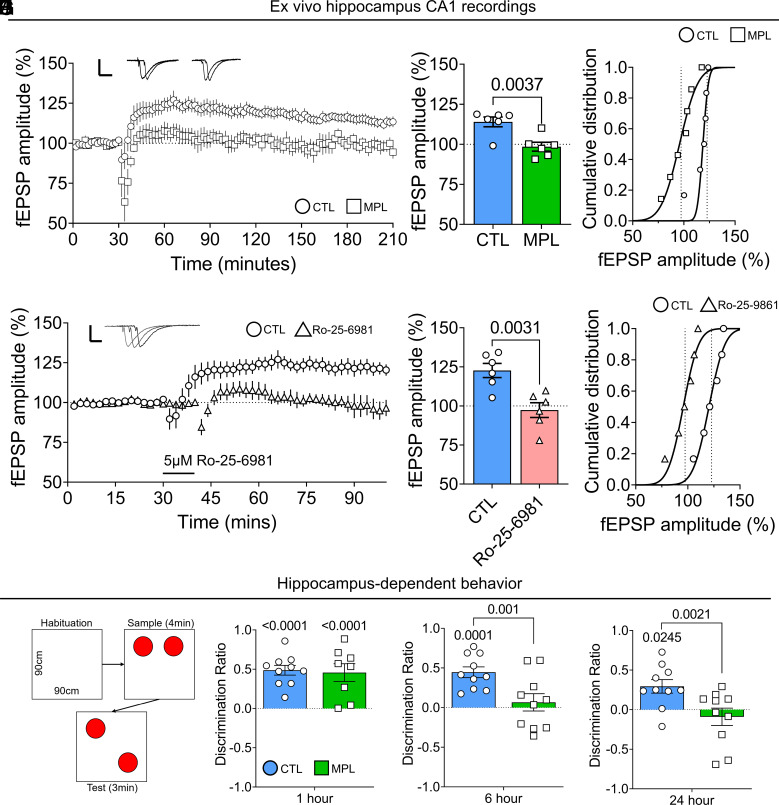
Methylprednisolone inhibits NMDAR-dependent LTP and impairs intermediate and long-term hippocampal-dependent memory formation. (*A*–*C*) LTP recorded during the early active phase for 3 h posthigh-frequency stimulation in the hippocampal slice from CTL and MPL-treated rats and quantified during a 30-min epoch 2.5 h post-HFS (n = 6 CTL active; n = 6 MPL active, *P* = 0.0037). (*D*–*F*) LTP recorded following 5 µM Ro-25-6981 application and HFS stimulation (n = 6 CTL active; n = 6 Ro-25-6981 active, *P* = 0.0031). (*G*) Schematic of object location memory (OLM) task. (*H*) OLM 1 h post acquisition phase in control and MPL-treated rats. *F*_1,32_ = 59.52, *P* < 0.0001, for learning, by two-way ANOVA. ****P < 0.0001. (*I*) OLM 6 h post acquisition phase in CTL and MPL-treated rats. *F*_1,36_ = 15.87, *P* = 0.0003, for memory, and *F*_1,36_ = 8.727, *P* = 0.0055, for treatment, by two-way ANOVA. ***P = 0.0001, ^###^*P* = 0.0010, Tukey’s multiple comparisons. (*J*) OLM 24 h post acquisition phase in CTL and MPL-treated rats. *F*_1,36_ = 7.646, *P* = 0.0089, for treatment, by two-way ANOVA. *P = 0.0245, ^##^*P* = 0.0021, Tukey’s multiple comparisons. Data are mean ± SEM. *P < 0.05.

CaMKII is essential for modulating LTP and spatial memory formation (56), and following the striking disruption to the dynamic expression of both N-methyl-D-aspartate (NMDA) receptor and CaMKII subunits, we show that hippocampal LTP and mPSCs were disrupted following MPL treatment. Again, using the same HFS protocol that was sensitive to regulation by time of day in control rats (Fig. 2 *E*–*I*), we found that MPL treatment abolished the formation of LTP during the early active period, when it could be readily elicited in controls (Fig. 4 *A*–*C*). Furthermore, in controls, LTP induction could be similarly blocked by application of the GluN2B-selective NMDAR antagonist, Ro-25-6981 (Fig. 4 *D*–*F*). This suggests that ablation of the circadian regulation of NMDARs and CaMKII results in disruption of downstream plasticity processes preventing their association at a time critical to nonaversive learning. Consistent with our data demonstrating corticosteroid sensitivity, NMDARs are critical in mediating the dendritic atrophy and synaptic dysfunction caused by chronic stress, (57, 58) with GluN2B appearing to play a pivotal role (59).

Following these robust effects of steroid treatment on the hippocampal transcriptome and synaptic physiology, we investigated whether MPL-induced disruption of NMDA receptor function had a consequent impact on hippocampal-dependent memory formation. After 5 d of MPL treatment, using a novel object location task, where training and testing were carried out during the early active period, short-term memory retrieval (1-h postacquisition phase) was unaffected (Fig. 4*H*). However, memory retrieval was impaired at intermediate (6-h) and long-term retrieval (24-h), postacquisition phase (Fig. 4 *I* and *J*). There was no difference in exploration time (*SI Appendix*, Fig. S4). Taken together, these data indicate that MPL treatment inhibits long-term, GluN2B-NMDAR dependent memory, but not short-term memory formation (60, 61).

## Discussion

Corticosteroids are widely used in clinical medicine to relieve signs and symptoms of many inflammatory and autoimmune disorders (62, 63). However, in addition to this, their use is often reported with cognitive and psychiatric symptoms—inducing a range of psychiatric adverse effects including depression, mania, and cognitive and memory impairment. These relationships have been known for decades, yet the mechanisms underlying these disturbances and their clinical management have been poorly described.

In this reverse translational study, we demonstrate that 5 d of corticosteroid treatment disrupts the rhythmic activity of hippocampal function through GRs, induces NMDAR-dependent synaptic dysfunction, and subsequently impairs memory. This model is supported by several lines of evidence: 1) Corticosteroids do not influence the master clock (SCN), or light/dark entraining behaviors due to a lack of GR expression in the SCN; 2) the hippocampus is rich in GRs, which in the presence of corticosteroids, bind to GREs on the essential clock gene *Per1* to mediate hippocampal activity in competition with central clock-mediated control; 3) synthetic corticosteroid treatment eliminates the circadian variation in circulating corticosteroids, and consequently the circadian variation in the activity of hippocampal GRs to influence clock gene expression and destabilize clock-entrained NMDAR/CamkII complexes; and 4) synthetic corticosteroid treatment blocks LTP, a cellular correlate of long-term memory, by disrupting NMDAR-dependent processes to impair long-term, but not short-term, hippocampal-dependent memory.

There are two regulators in normal chronophysiology. On the one hand, the SCN is the master regulator of circadian timing, entraining behaviors such as feeding, sleep, and memory processes to ~24 h (64). On the other hand, extra-SCN oscillators, such as those in the hippocampus and other forebrain regions, are entrained by SCN-dependent neural and humoral inputs (65). Pertinent to this study, one of the more powerful humoral signals is from adrenal corticosteroids (66), which exhibit a robust circadian secretory rhythm that peaks prior to wakening (2) in preparation for the neurocognitive activities that ensue. Synthetic long-acting corticosteroids, such as MPL, are therefore poised to mediate influence over these endogenous systems in the presence of the master clock.

Indeed, corticosteroids exert vast and variable effects on the body’s physiology, both peripherally and centrally (2, 67, 68). Consistent with previous studies, we found that MPL treatment suppressed appetite, and subsequently decreased body weight (*SI Appendix*, Fig. S1 *D*–*F*). However, interestingly, we identified distinct and specific actions of MPL on the brain. For instance, the SCN remained protected from MPL-mediated disruption, as previously reported with dexamethasone (69). However, the GR-rich hippocampus was sensitive to corticosteroid interference (70). The identification of GREs upstream of the *Per1* gene, and its corticosteroid-induced transcription, underpins a mechanism whereby GR-expressing extra-SCN oscillators can be disturbed by corticosteroids, and may account for the loss of circadian rhythms often seen in hypercortisolemic states of Cushing’s disease and syndrome (71). Notably, our RNAseq dataset which encompassed whole hippocampus found that *Per1* mRNA expression was up-regulated throughout the day with MPL treatment. Interestingly, *Per1*^(−/−)^ mice also exhibit spatial memory deficits. These targeted manipulations of *Per1* are opposing (i.e., overexpression vs. knockout) yet led to a similar outcome, suggesting that any disruption to the rhythmic expression of *Per1* may be detrimental to spatial memory performance.

It is well established that corticosteroids are secreted from the adrenal gland in a circadian pattern with underlying ultradian pulses, and common to several neuropsychiatric disorders are HPA axis hyperactivity or steroid therapy (72–74). We found that 5 d of MPL treatment induced a prolonged increase in activated nuclear GR levels throughout the full circadian cycle and disrupted the circadian expression of NMDAR and CAMKII subunits. Both NMDAR and CAMKII are known to be essential mediators of neural plasticity (75), a process that is causally linked with the successful encoding of mnemonic information (76). Further to this, CAMKII is essential for coupling time of day to behavioral rhythms (33) and improved memory performance (77).

Consistent with our findings, other synthetic corticosteroids, particularly dexamethasone, can impair memory (78). However, translational studies often assess memory performance during the early inactive period, when endogenous circulating corticosteroids are low, therefore not time relevant to biological rhythms (79) or peak cognitive performance of the participants. Similarly, many rodent studies assess both hippocampal synaptic plasticity processes and memory performance during the animals’ inactive period, leading to the somewhat incomplete conclusion that effective memory consolidation requires a strong stress association. These tests use aversive or fear conditioning-based tasks such as inescapable foot shock, which induces a robust stress response in the animal, resulting in similar elevated glucocorticoid levels to those circulating at the onset of the active phase. Consistent with our interpretation that elevated glucocorticoids during the inactive phase can influence the molecular physiology of the brain, acute administration of corticosteroids has been demonstrated to enhance memory consolidation during nonaversive memory tasks performed during rats’ inactive phase. (80, 81). The specificity of corticosteroid, duration of action, as well as the timing and type of experimental testing, may contribute to differences in corticosteroid-mediated memory formation in the literature. Importantly in this study, we have identified a mechanism involving dysregulation of the hippocampal circadian clock, in which neurocognitive decline is mediated by a commonly prescribed synthetic corticosteroid. A limitation of this work is that a rescue strategy is yet to be established, and this will require future investigation. Moreover, how various corticosteroids (endogenous and synthetic) contribute to such differential outcomes on hippocampal function warrants further investigation. This study also used male rats, and therefore does not consider the action of corticosteroids on circadian processes and memory performance in females. Indeed, it is well established that in hippocampal CA1, LTP is dependent on locally synthesized estrogen (82), and that blocking estradiol synthesis disrupts LTP in females only (82, 83). Therefore, understanding how endogenous and exogenous corticosteroids may interact with and disrupt these processes warrants investigation.

Nevertheless, our ex vivo physiology data suggest that time of day is a potent modulator of neuronal activity and plasticity processes in CA1 neurons. Crucially, we show that this circadian regulation is modified by long-acting corticosteroid treatment, disrupting the dynamic control of miniature postsynaptic currents (mPSCs) and preventing LTP induction. These data support the notion that circadian and ultradian corticosteroid fluctuations play an important role in maintaining the rhythmic activity of the hippocampus via dynamic transcription of target genes. This has major physiological consequences considering the importance of mPSCs in the maintenance of synaptic connections and dendritic spines (84), as long-term alterations to these dynamics can result in critical changes to hippocampal circuitry and function, as demonstrated by the prevention of hippocampal-dependent memory formation (Fig. 4).

In summary, a large proportion of patients prescribed with corticosteroids report cognitive decline and memory impairment (16). Our data reveal a GR-mediated pathway that underlies the circadian regulation of hippocampal-dependent memory formation which is vulnerable to long-acting corticosteroid treatment. Currently, there are no clinical guidelines for treating corticosteroid-induced adverse effects. It is, perhaps, quite surprising that the brain-specific effects of such treatment has had relatively little scientific investigation given the broad application, widespread clinical use, and adverse psychiatric outcomes (15, 85, 86). Advancing our knowledge of corticosteroid-mediated regulation of hippocampal function will take us a step closer to understanding the mechanisms underpinning several prevalent major mental illnesses and suggest tailoring treatment regimens for prevention and intervention of corticosteroid (endogenous and synthetic)-induced disorders.

## Methods

Adult male Lister hooded rats (250 to 350 g, 9 to 11 wk) (Envigo, UK) were used in all experimental procedures. Male rats were used in this study to limit estrous cycle influence on corticosterone release (87). Animals were maintained in standard housing conditions under a 12:12 gradual light/dark cycle in sound-attenuating rooms. A red light was used during the night phase to allow researchers to manipulate the animals. During the night phase, no external light could enter the room when opening the door. Food and water were available ad libitum. Throughout all experimental procedures, the same researcher took care of the animals to limit stress-induced effects and was blinded to treatment groups. All procedures were carried out in accordance with the UK Animals (Scientific Procedures) Act 1986 under PPL 30/3114 and PIL I04092F5F.

### Adrenalectomy and Jugular Vein Cannulation.

For studies identifying glucocorticoid-specific control of hippocampal gene transcription (*SI Appendix*, Fig. S4), rats received balanced anesthesia using veterinary isoflurane (Merial Animal Health, Woking, UK) prior to bilateral adrenalectomy and cannulation of the right jugular vein for infusion of corticosterone. Rats recovered for 5 d postsurgery on 15 µg/mL corticosterone in 0.9% saline drinking solution to maintain isotonic levels. This solution was replaced 12 h prior to experiments with 0.9% saline to ensure washout of circulating corticosterone.

### Corticosterone Infusion.

Rats received either a 30-min infusion of corticosterone (0.75 mg/mL corticosterone-2-hydroxypropyl-*β-*cyclodextrin; Sigma-Aldrich, Gillingham, UK) dissolved in sterile 0.45% (w/v) NaCl, or sterile 0.45% (w/v) NaCl at ZT2. A New Era NE-1800 computer-driven infusion pump (World Precision Instruments, Aston, UK) delivered 1 mL/h for 30 min via the indwelling jugular cannula. Rats were killed 2 h post infusion start at ZT4 (*SI Appendix*, Fig. S4).

### MPL Treatment.

For all studies assessing the role of MPL, rats (randomly assigned to treatment) were given MPL sodium succinate (Solu-Medrol; Pharmacia Ltd, Sandwich, UK) 1 mg/mL ad libitum in drinking water for 5 d (prolonged treatment protocol), prior to the start of all further procedures. Concentrations of MPL in drinking water (1 mg/mL) were optimized in previous experiments as the minimum dose required to reproducibly induce hippocampal GR activation and suppress endogenous corticosterone (20 mg/day). Body weight was monitored throughout all procedures. Ad libitum access to MPL in drinking water was designed to limit any external stimuli that may act as a zeitgeber (88) and also represents greater than 1% of the human population that are prescribed oral glucocorticoid treatment (15). At this dose, no difference in fluid consumption was observed across days, and circulating endogenous corticosteroids (measured at the end of 5-d treatment) were suppressed (*SI Appendix*, Fig. S3).

### Radioimmunoassay.

Using an automated gamma counter (PerkinElmer, US), a corticosterone radioimmunoassay measured endogenous levels in blood samples collected immediately following rapid decapitation. An 11-point standard curve of known corticosterone concentrations was prepared in B-buffer (25 mM tri-sodium citrate, 50 mM sodium dihydrogen orthophosphate, 1 mg/mL bovine serum albumin: pH3). Plasma obtained was diluted in triplicate at a ratio of either 1:10 or 1:50 in B-buffer. A specific corticosterone antibody (kindly provided by G. Makara, Institute of Experimental Medicine, Budapest, Hungary) was diluted at a ratio of 1:50 in B-buffer and 50 μL added to 100 μL standards, unknown samples, and Quality Control (QC20 and QC100) tubes. Tracer (Izotop, Institute of Isotopes, Hungary) was diluted in B-buffer to give total counts of 3,750 cpm in 50 μL and added to all tubes (50 μL/tube). Tubes were incubated overnight at 4 °C. Charcoal suspension (5 g charcoal added to 0.5 g dextran T70 dissolved in 1L B-buffer) was prepared and 500 μL was added to all tubes and briefly vortexed. Blocks were centrifuged at 4,000 rpm at 4 °C and the resulting supernatant was aspirated off. Unknown samples were determined from interpolation of the standard curve.

### In Situ Hybridization Histochemistry.

Whole brains were cryosectioned into coronal 12 μm sections, and thaw mounted on gelatin/chrome alum-coated slides. The location of the SCN was determined according to coordinates in the rat brain atlas (89). ^35^S 3′- end-labeled deoxyoligonucleotide complementary to transcripts encoding *Per1* (5′- CTC TTG TCA GGA GGA ATC CGG GGA GCT TCA TAA CCA GAG TGG ATG -3′), *Per2* (5′- GTG GCC TTC CGG GAT GGG ATG TTG GCT GGG AAC TCG CAC TTT CTT -3′), and *hnAVP* (5′- GCA CTG TCA GCA GCC CTG AAC GGA CCA CAG TGG TAC -3′) was used. The in situ hybridization protocol has been previously described in detail (90). Briefly, sections were fixed in 4 % (w/v) formaldehyde for 5 min and incubated in saline containing 0.25 % (v/v) acetic anhydride and 0.1 M triethanolamine for 10 min. Sections were then dehydrated in ethanol, delipidated in chloroform, and partially rehydrated. Hybridization with a total count of 1 × 10^6^ cpm was performed overnight at 37 **°**C in 45 μL hybridization buffer under Nescofilm (Bando Kagaku, Osaka, Japan). After hybridization, sections were washed 4 times with SSC (150 mM NaCl and 15 mM sodium citrate) for 1 h at 65 °C and for an additional hour with two changes of SSC at room temperature. Hybridized sections were exposed for autoradiography (Hyperfilm, Amersham, Bucks, UK) for 1 wk. The amount of bound probe was analyzed in comparison to ^14^C-labeled standards (Amersham, Bucks, UK) using image analysis software (NIH Image 1.6.2, W. Rasband, NIH, Bethesda, MD, USA). The obtained results were represented in arbitrary units setting the mean optical density (OD) obtained from sham-operated rats.

### Chromatin Immunoprecipitation (ChIP).

Hippocampi were fixed for ChIP and processed to chromatin in sodium dodecyl sulfate (SDS) lysis buffer [2% SDS, 10 mM EDTA, 50 mM Tris-HCl (pH 8.1)] as previously described (91). Chromatin was sheared with a Sonifier 450 (Branson Ultrasonics, Danbury, CT) using 4× 10-s pulses at 10% output, and then cleared of cellular debris by centrifugation. For each IP, chromatin was diluted 1:10 in ChIP dilution buffer [167 mM NaCl, 16.7 mM Tris-HCl (pH 8.1), 1.1% Triton X-100, 1.2 mM EDTA, 0.01% SDS] supplemented with complete protease inhibitor (Sigma). Reactions were immunoprecipitated overnight at 4 °C with 2 µg anti-GR M-20X (Santa Cruz Biotechnology, US) or rabbit nonimmune serum (2µg sc-2027; Santa Cruz, USA) for the negative control. GR–DNA complexes were collected onto protein A–conjugated Dynabeads (Invitrogen, Paisley, UK) and washed to remove nonspecific binding. Purified DNA was resuspended in nuclease-free water (Ambion, Huntington, UK).

PCR primers (forward: 5′- CCAAGGCTGAGTGCATGTC -3′; reverse: 5′- GCGGC​CAGCGCACTA -3′) were designed to amplify across a previously described GRE (20, 91) in the rat Period 1 gene promoter. Samples were amplified with Sybr Green master mix (Applied Biosystems) in accordance with the manufacturer’s instructions. GR binding for each sample was calculated relative to 1% input chromatin taken from each individual sample, using the %Input method, described in (ThermoFisher Scientific, http://bit.ly/ChIPAnalysisTFS).

### RNA and qPCR.

Animals were anesthetized with isoflurane in the animal facility, and eight were killed every 4 h (six time points). The brain was quickly extracted, and the hippocampus was removed and rapidly frozen in liquid nitrogen. The time between decapitation and sample freezing was <1 min to limit RNA degradation. The collection of the hippocampal tissue per time point was <20 min (10 min either side of the hour mark).

Total RNA was extracted from frozen whole hippocampi using the miRNeasy total RNA extraction kit protocol (Qiagen, US) following the manufacturer’s guide and included a DNase digestion step. Samples were stored at −80 °C. All samples were assessed for RNA quality and quantity using a Nanodrop (ThermoScientific, US). Samples sent for RNAseq were further assessed for RNA integrity on the 2200 TapeStation system (Agilent, US). Sequenced samples had >8.0 RIN score.

Each PCR contained 1 µL cDNA, with a total volume of 10 µL. qPCR runs consisted of an initial 95 °C holding stage for 20 s, followed by 40 cycles of 95 °C (1 s) and 60 °C (20 s), followed by a melt curve step, consisting of 40 cycles of 95 °C (15 s) and 60 °C (1 min), with a final denaturing step of 95 °C (15 s) using a StepOnePlus PCR machine (Applied Biosystems, Life Technologies, UK).

### Whole-Genome RNAseq and Analyses.

RNAseq of hippocampal tissue was carried out using TruSeq Stranded Total RNA kit and protocols (Illumina, US). A 1 μg aliquot total RNA from each hippocampus was prepared for RNAseq with 48 samples in total. First, bioanalyzer traces were carried out on all samples for RNA quantity and quality check. RIN scores above 8.0 were deemed high enough quality to take through to RNAseq. Following library preparation, the samples were run on a HiSeq 2500 machine (Illumina, US). Eight samples were run in each lane, in a paired-end sequencing run, generating approximately 300 million reads across the eight samples.

High-throughput RNAseq raw data were uploaded to Galaxy (92), an open-access portal for next-generation sequencing analysis. For the circadian sequencing experiments, an N of 4 was used in each group for a total of 48 whole hippocampi samples, and three lanes of data were collected for each sample. For the acute corticosterone administration experiments, an N of 4 was used in each group for a total of 8 whole hippocampi samples. Sequencing files were aligned to the *Rattus norvegicus* (Rn6) genome-generating individual Binary Alignment Map (BAM) files. These BAM files from each of the three lanes were merged. The merged BAM files were analyzed for gene expression differences using Tophat2, Cufflinks, and CuffDiff analyses aligning to the Rn6 genome (93). The parameters included geometric library organization and pooled dispersion estimation, and the false discovery rate was set at 0.05. Minimum alignment count—10, multi-read correct, bias correction and Cufflinks effective length correction were also included. Differential expression was assessed across time using CuffDiff version 15 (2020-06-16), with all time points compared to ZT 2. Any gene that failed DE analysis because of low gene expression [based on minimum alignment count (94, 95)] at any time point was removed from further analyses. Therefore, in the circadian RNAseq analysis–vehicle-treated rats, DE analysis was carried out on 13,939 genes. In MPL-treated rats, DE analysis was carried out on 16,269 genes. In the acute corticosterone administration to ADX rats, DE analysis was carried out on 16,266 genes. Differential gene expression was calculated from fragments per kilobase per million mapped reads values. Multiple hypothesis correction was carried out using the Benjamini–Hochberg test. Data deemed statistically significant, FDR < 0.05.

The Database for Annotation, Visualization and Integrated Discovery (96) was used to determine GO and pathways. GO categories tested included biological processes and pathways from the Kyoto Encyclopedia of Genes and Genomes (KEGG).

### RNAseq Rhythmicity Analysis.

For CTL and MPL-treated groups, we focused on 24 h periodicity using harmonic regression on the log2 transformed signals, as previously described (52), from gene expression data sampled every 4 h. Each hour on the clock face refers to the total number of genes that peak at that time of day.

### Brain Slice Preparation.

The brain was rapidly removed at either ZT0030 (30 to 60 min following light change–inactive phase recording) or ZT1230 (30 to 60 min following light change–active phase recording) (*SI Appendix*, Fig. S5). These times were chosen based upon our RNAseq data. Peak Per1 mRNA expression in controls was between ZT 10 and ZT 14. Nadir Per1 mRNA expression in controls was between ZT 22 and ZT 2. A minimum of 2 h after peak and trough expression was chosen to allow for functional protein availability. Slices placed in ice-cold slicing solution contained (in mM): 52.5 NaCl, 2.5 KCl, 25 NaHCO_3_, 1.25 NaH_2_PO_4_, 5 MgCl_2_, 25 D-Glucose, 100 sucrose, 2 CaCl_2_, 0.1 kynurenic acid, bubbled with 95% O_2_/5% CO_2_. Parasagittal slices (400 μm) were cut using a vibrating blade microtome (Leica VT1000 S) while maintained in slicing solution. The hippocampus was then isolated and placed in a holding chamber containing artificial cerebrospinal fluid (aCSF) (containing (in mM) 124 NaCl, 3 KCl, 26 NaHCO_3_, 1.4 NaH_2_PO_4_, 1 MgSO4, 10 D-Glucose, 2 CaCl_2_) where it was incubated at 32 °C for 30 min, followed by RT for at least 30 min (for whole-cell recordings), or kept at room temperature for at least 1 h (for field electrophysiology) before being transferred to the recording chamber.

### Extracellular Electrophysiology.

Hippocampal slices were placed in a submersion style recording chamber maintained at 30 °C and continuously perfused with oxygenated aCSF at a flow rate of ~2 mL/min. Field excitatory postsynaptic potentials were evoked at 0.033 Hz by placing bipolar stimulating electrodes on the Schaffer collateral fibers with the recording electrode positioned in CA1 stratum radiatum. Recording electrodes were prepared by pulling borosilicate capillary tubes with a P-97 Flaming/Brown micropipette puller (Sutter Instrument Co) to a tip resistance of 2 to 6 MΩ and were then back-filled with aCSF. Signals were amplified using an AxoClamp 2B amplifier (Molecular Devices), digitized using a BNC-2110 (National Instruments) board, and 50/60 Hz noise eliminated by a Hum Bug (Quest Scientific). Data were acquired and analyzed using WinLTP software (97). Signals were averaged over a period of 2 min and a stable baseline recording of 30 min was acquired before LTP induction by delivery of 10 Hz stimulation for 90 s.

### Whole-Cell Electrophysiology.

Whole-cell recordings were taken from pyramidal neurons in the CA1 cell layer. Borosilicate pipettes (2 to 6 MΩ) were filled with an internal solution containing (in mM): 8 NaCl, 130 CsMeSO_4_, 10 HEPES, 0.5 EGTA, 4 MgATP, 0.3 NaGTP, and 5 QX-314. Recordings were accepted for analysis with an uncompensated series resistance of <2.5 times the pipette resistance. Recordings were not corrected for series resistance due to the small current amplitudes. During mEPSC and mIPSC recordings, 100 µM D-AP5 was added to the perfused aCSF to block NMDA receptor-mediated currents. mEPSCs were recorded at a membrane potential of −70 mV and for mIPSCs, membrane potential was held at 0 mV. Recordings were amplified using an AxoClamp 700B (Molecular Devices) for whole-cell voltage-clamp recordings. Data were acquired using WinLTP software at a sampling rate of 10 KHz, and filtered at 6 KHz, before being analyzed offline using ClampFit 9.2. mEPSCs and mIPSCs were identified when the rise time was faster than the decay time and had a peak amplitude >6 mV.

### Object Location Memory (OLM) Task.

For all memory testing, different rats were used at all time points and treatment. Rats were transferred to a sound-attenuating behavior room in low light (40 to 50 lx on arena floor) at ZT11.

Rats were left to habituate to the room for at least an hour, prior to starting behavioral experiments. Similarly to our electrophysiology studies, this time was chosen based upon our RNAseq data. Peak Per1 mRNA expression in controls was between ZT 10 and ZT 14. A minimum of 2 h after peak expression was chosen to allow for functional protein availability. Rats were handled for 1 wk in the behavior room prior to experimental start, followed by habituation to the arena without stimuli for 10 min daily for 5 d. Training and testing occurred in an open-top arena (50 × 90 × 100 cm) made of wood. The walls inside the arena were black and floor covered in sawdust. An overhead camera recorded behavior for analysis. Exploration was scored when the rat head orientated toward the object and came within 1 cm of the object. The objects were constructed from Duplo blocks, which were too heavy for the animals to displace. During training, rats were exposed to two identical objects and allowed to explore for 4 min. These objects were placed in the far side of the arena, 10 cm away from the walls, to allow full access around the objects. During the retention test (1 h for short-term memory, 6 h for intermediate memory, or 24 h for long-term memory), rats were allowed to explore for 3 min. During testing, one of the objects was moved to a new location. The position of the object was counterbalanced between rats. Total exploration time was recorded, and preference for the novel item was expressed as a discrimination index.

### Activity and Temperature Recording.

All rats were individually housed for telemetry recordings for technical reasons. Rats were implanted intraperitoneally with telemeters (PTD 4000 E-mitter, Starr Life Sciences Corp, US). Following recovery (>3 d), cages were placed upon receivers (ER-4000 receiver, Starr Life Sciences Corp, US). Locomotor activity and core body temperature data were collected every 10 min for five consecutive days. Data collected were analyzed with R CRAN package *cosinor* to measure bathyphase, acrophase, and circadian period under a 24-h period.

### Statistical Analyses.

Results are presented as mean ± SEM. All statistical analyses were performed using GraphPad Prism (v 9.1, GraphPad software Inc., US), with parametric and nonparametric tests used where appropriate. Details of specific tests are provided in the figure legends. Statistical significance was set at *P* < 0.05.

## Supplementary Material

Appendix 01 (PDF)

Dataset S01 (XLSX)

Dataset S02 (XLSX)

Dataset S03 (XLSX)

Dataset S04 (XLSX)

Dataset S05 (XLSX)

Dataset S06 (XLSX)

## Data Availability

All RNAseq data is deposited and available at Gene Expression Omnibus with accession numbers GSE223761 (MPL) (98) and GSE224038 (acute corticosterone) (99). All study data are included in the article and/or *SI Appendix*.

## References

[1] R. Refinetti, M. Menaker, The circadian rhythm of body temperature. Physiol. Behav. **51**, 613–637 (1992). 1523238 10.1016/0031-9384(92)90188-8

[2] S. L. Lightman, M. T. Birnie, B. L. Conway-Campbell, Dynamics of ACTH and cortisol secretion and implications for disease. Endocr. Rev. **41**, 470–490 (2020). 10.1210/endrev/bnaa002 PMC7240781 32060528

[3] S. Panda, Circadian physiology of metabolism. Science **354**, 1008–1015 (2016). 27885007 10.1126/science.aah4967 PMC7261592

[4] F. Fernandez , Dysrhythmia in the suprachiasmatic nucleus inhibits memory processing. Science **346**, 854–857 (2014). 25395537 10.1126/science.1259652 PMC4459503

[5] E. D. Herzog, T. Hermanstyne, N. J. Smyllie, M. H. Hastings, Regulating the suprachiasmatic nucleus (SCN) circadian clockwork: Interplay between cell-autonomous and circuit-level mechanisms. Cold Spring Harb. Perspect. Biol. **9**, a027706 (2017). 28049647 10.1101/cshperspect.a027706 PMC5204321

[6] K. J. Debski , The circadian dynamics of the hippocampal transcriptome and proteome is altered in experimental temporal lobe epilepsy. Sci. Adv. **6**, eaat5979 (2020). 33036982 10.1126/sciadv.aat5979 PMC10764101

[7] A. Jilg , Temporal dynamics of mouse hippocampal clock gene expression support memory processing. Hippocampus **20**, 377–388 (2010). 19437502 10.1002/hipo.20637

[8] L. M.-C. Wang , Expression of the circadian clock gene *Period2* in the hippocampus: Possible implications for synaptic plasticity and learned behaviour. ASN Neuro **1**, e00012 (2009). 19570032 10.1042/AN20090020 PMC2695588

[9] S. M. Wardlaw, T. X. Phan, A. Saraf, X. Chen, D. R. Storm, Genetic disruption of the core circadian clock impairs hippocampus-dependent memory. Learn. Mem. **21**, 417–423 (2014). 25034823 10.1101/lm.035451.114 PMC4105720

[10] C. M. Pariante, S. L. Lightman, The HPA axis in major depression: Classical theories and new developments. Trends Neurosci. **31**, 464–468 (2008). 18675469 10.1016/j.tins.2008.06.006

[11] A. Tsui, M. Richards, A. Singh-Manoux, C. Udeh-Momoh, D. Davis, Longitudinal associations between diurnal cortisol variation and later-life cognitive impairment. Neurology **94**, e133–e141 (2020). 31831603 10.1212/WNL.0000000000008729 PMC6988984

[12] N. Amasi-Hartoonian, L. Sforzini, A. Cattaneo, C. M. Pariante, Cause or consequence? Understanding the role of cortisol in the increased inflammation observed in depression. Curr. Opin. Endocr. Metab. Res. **24**, 100356 (2022). 35634363 10.1016/j.coemr.2022.100356 PMC7612780

[13] K. Kalafatakis , Ultradian rhythmicity of plasma cortisol is necessary for normal emotional and cognitive responses in man. Proc. Natl. Acad. Sci. U.S.A. **115**, E4091–E4100 (2018). 29632168 10.1073/pnas.1714239115 PMC5924881

[14] S. Rohatagi , Pharmacokinetics of methylprednisolone and prednisolone after single and multiple oral administration. J. Clin. Pharmacol. **37**, 916–925 (1997). 9505983 10.1002/j.1552-4604.1997.tb04266.x

[15] R. A. Overman, J.-Y. Yeh, C. L. Deal, Prevalence of oral glucocorticoid usage in the United States: A general population perspective. Arthritis Care Res. (Hoboken) **65**, 294–298 (2013). 22807233 10.1002/acr.21796

[16] P. Keenan, M. Jacobson, R. Soleymani, M. Stress, D. Yaldoo, The effect on memory of chronic prednisone treatment in patients with systemic disease. Am. Acad. Neurol. **47**, 1396–1402 (1996). 10.1212/wnl.47.6.1396 8960717

[17] E. S. Brown, J. Wolfshohl, M. U. Shad, M. Vazquez, I. J. Osuji, Attenuation of the effects of corticosteroids on declarative memory with lamotrigine. Neuropsychopharmacology **33**, 2376–2383 (2008). 18004283 10.1038/sj.npp.1301627 PMC3238801

[18] J. P. Mersky, J. Topitzes, A. J. Reynolds, Impacts of adverse childhood experiences on health, mental health, and substance use in early adulthood: A cohort study of an urban, minority sample in the U.S. Child Abus. Negl. **37**, 917–925 (2013). 10.1016/j.chiabu.2013.07.011 PMC4090696 23978575

[19] W. Nakamura, S. Yamazaki, N. N. Takasu, K. Mishima, G. D. Block, Cellular/molecular differential response of period 1 expression within the suprachiasmatic nucleus. Neuroscience **25**, 5481–5487 (2005). 15944376 10.1523/JNEUROSCI.0889-05.2005 PMC6724974

[20] T. E. Reddy, J. Gertz, G. E. Crawford, M. J. Garabedian, R. M. Myers, The hypersensitive glucocorticoid response specifically regulates period 1 and expression of circadian genes. Mol. Cell. Biol. **32**, 3756–3767 (2012). 22801371 10.1128/MCB.00062-12 PMC3430195

[21] J. M. Reul, E. R. Kloet, Two receptor systems for corticosterone in rat brain: Microdistribution and differential occupation. Endocrinology **117**, 2505–2511 (1985). 2998738 10.1210/endo-117-6-2505

[22] J. P. Herman, R. Spencer, Regulation of hippocampal glucocorticoid receptor gene transcription and protein expression in vivo. J. Neurosci. **18**, 7462–7473 (1998). 9736665 10.1523/JNEUROSCI.18-18-07462.1998 PMC6793224

[23] P. Kitchener, F. Di Blasi, E. Borrelli, P. V. Piazza, Differences between brain structures in nuclear translocation and DNA binding of the glucocorticoid receptor during stress and the circadian cycle. Eur. J. Neurosci. **19**, 1837–1846 (2004). 15078557 10.1111/j.1460-9568.2004.03267.x

[24] E. J. Waite , Ultradian corticosterone secretion is maintained in the absence of circadian cues. Eur. J. Neurosci. **36**, 3142–3150 (2012). 22823558 10.1111/j.1460-9568.2012.08213.x

[25] R. Salgado-Delgado, M. Angeles-Castellanos, N. Saderi, R. M. Buijs, C. Escobar, Food intake during the normal activity phase prevents obesity and circadian desynchrony in a rat model of night work. Endocrinology **151**, 1019–1029 (2010). 20080873 10.1210/en.2009-0864

[26] C. Liston , Circadian glucocorticoid oscillations promote learning-dependent synapse formation and maintenance. Nat. Neurosci. **16**, 698–705 (2013). 23624512 10.1038/nn.3387 PMC3896394

[27] Y. Takahashi, K. Sawa, T. Okada, The diurnal variation of performance of the novel location recognition task in male rats. Behav. Brain Res. **256**, 488–493 (2013). 24008072 10.1016/j.bbr.2013.08.040

[28] H. J. Hoffmann, D. Balschun, Circadian differences in maze performance of C57BI/6 Ola mice. Behav. Processes **27**, 77–83 (1992). 24924493 10.1016/0376-6357(92)90017-8

[29] V. S. Valentinuzzi, L. Menna-Barreto, G. F. Xavier, Effect of circadian phase on performance of rats in the morris water maze task. J. Biol. Rhythms **19**, 312–324 (2004). 15245650 10.1177/0748730404265688

[30] W. Hauber, A. Bareiß, Facilitative effects of an adenosine A1/A2 receptor blockade on spatial memory performance of rats: Selective enhancement of reference memory retention during the light period. Behav. Brain Res. **118**, 43–52 (2001). 11163632 10.1016/s0166-4328(00)00307-7

[31] K. H. Snider, K. A. Sullivan, K. Obrietan, Circadian regulation of hippocampal-dependent memory: Circuits, synapses, and molecular mechanisms. Neural Plast. **2018**, 7292540 (2018). 29593785 10.1155/2018/7292540 PMC5822921

[32] J. R. Pooley , Genome-wide identification of basic helix–loop–helix and NF-1 motifs underlying GR binding sites in male rat hippocampus. Endocrinology **158**, 1486–1501 (2017). 28200020 10.1210/en.2016-1929 PMC5460825

[33] N. Kon , CaMKII is essential for the cellular clock and coupling between morning and evening behavioral rhythms. Genes Dev. **28**, 1101–1110 (2014). 24831701 10.1101/gad.237511.114 PMC4035538

[34] O. Rawashdeh, A. Jilg, E. Maronde, J. Fahrenkrug, J. H. Stehle, *Period1* gates the circadian modulation of memory-relevant signaling in mouse hippocampus by regulating the nuclear shuttling of the CREB kinase pP90RSK. J. Neurochem. **138**, 731–745 (2016). 27246400 10.1111/jnc.13689

[35] L. Mikasova , Stress hormone rapidly tunes synaptic NMDA receptor through membrane dynamics and mineralocorticoid signalling. Sci. Rep. **7**, 8053 (2017). 28808323 10.1038/s41598-017-08695-3 PMC5556050

[36] H. M. Chao, P. H. Choo, B. S. McEwen, Glucocorticoid and mineralocorticoid receptor mRNA expression in rat brain. Neuroendocrinology **50**, 365–371 (1989). 2554175 10.1159/000125250

[37] A.Y.-L. So, T. U. Bernal, M. L. Pillsbury, K. R. Yamamoto, B. J. Feldman, Glucocorticoid regulation of the circadian clock modulates glucose homeostasis. Proc. Natl. Acad. Sci. U.S.A. **106**, 17582–17587 (2009). 19805059 10.1073/pnas.0909733106 PMC2757402

[38] P. Gómez-Abellán , Glucocorticoids affect 24 h clock genes expression in human adipose tissue explant cultures. PLoS One **7**, e50435 (2012). 23251369 10.1371/journal.pone.0050435 PMC3519463

[39] B. Roozendaal, Stress and memory: Opposing effects of glucocorticoids on memory consolidation and memory retrieval. Neurobiol. Learn. Mem. **78**, 578–595 (2002). 12559837 10.1006/nlme.2002.4080

[40] E. Y. Yuen , Acute stress enhances glutamatergic transmission in prefrontal cortex and facilitates working memory. Proc. Natl. Acad. Sci. U.S.A. **106**, 14075–14079 (2009). 19666502 10.1073/pnas.0906791106 PMC2729022

[41] D. Chaudhury, C. S. Colwell, Circadian modulation of learning and memory in fear-conditioned mice. Behav. Brain Res. **133**, 95–108 (2002). 12048177 10.1016/s0166-4328(01)00471-5

[42] L. C. Lyons, C. L. Green, A. Eskin, Intermediate-term memory is modulated by the circadian clock. J. Biol. Rhythms **23**, 538–542 (2008). 19060262 10.1177/0748730408325359 PMC2747098

[43] L. C. Lyons, O. Rawashdeh, A. Katzoff, A. J. Susswein, A. Eskin, Circadian modulation of complex learning in diurnal and nocturnal Aplysia. Proc. Natl. Acad. Sci. U.S.A. **102**, 12589–12594 (2005). 16116090 10.1073/pnas.0503847102 PMC1194922

[44] H. J. Gritton, A. M. Stasiak, M. Sarter, T. M. Lee, Cognitive performance as a zeitgeber: Cognitive oscillators and cholinergic modulation of the SCN entrain circadian rhythms. PLoS One **8**, e56206 (2013). 23441168 10.1371/journal.pone.0056206 PMC3575350

[45] R. A. Sarabdjitsingh , Ultradian corticosterone pulses balance glutamatergic transmission and synaptic plasticity. Proc. Natl. Acad. Sci. U.S.A. **111**, 14265–14270 (2014). 25225407 10.1073/pnas.1411216111 PMC4191766

[46] J. C. Buurstede , Hippocampal glucocorticoid target genes associated with enhancement of memory consolidation. Eur. J. Neurosci. **55**, 2666–2683 (2022), 10.1111/ejn.15226. 33840130 PMC9292385

[47] K. Mizoguchi , Chronic stress induces impairment of spatial working memory because of prefrontal dopaminergic dysfunction. J. Neurosci. **20**, 1568–1574 (2000). 10662846 10.1523/JNEUROSCI.20-04-01568.2000 PMC6772382

[48] D. Stavreva , Ultradian hormone stimulation induces glucocorticoid receptor-mediated pulses of gene transcription. Nat. Cell Biol. **11**, 1093–1102 (2009). 19684579 10.1038/ncb1922 PMC6711162

[49] D. G. Dillon, D. A. Pizzagalli, Mechanisms of memory disruption in depression. Trends Neurosci. **41**, 137–149 (2018). 29331265 10.1016/j.tins.2017.12.006 PMC5835184

[50] K. Maiese, Neurodegeneration, memory loss, and dementia: The impact of biological clocks and circadian rhythm. Front. Biosci. **26**, 614 (2021). 10.52586/4971 PMC8756734 34590471

[51] D. Coluccia , Glucocorticoid therapy-induced memory deficits: Acute versus chronic effects. J. Neurosci. **28**, 3474–3478 (2008). 18367613 10.1523/JNEUROSCI.4893-07.2008 PMC6670588

[52] J. Wang , Circadian clock-dependent and -independent posttranscriptional regulation underlies temporal mRNA accumulation in mouse liver. Proc. Natl. Acad. Sci. U.S.A. **115**, E1916–E1925 (2018). 29432155 10.1073/pnas.1715225115 PMC5828596

[53] M. A. Busche , Rescue of long-range circuit dysfunction in Alzheimer’s disease models. Nat. Neurosci. **18**, 1623–1630 (2015). 26457554 10.1038/nn.4137

[54] M. A. Busche, A. Konnerth, Impairments of neural circuit function in Alzheimer’s disease. Philos. Trans. R. Soc. Lond. B Biol. Sci. **371**, 20150429 (2016). 27377723 10.1098/rstb.2015.0429 PMC4938029

[55] H. Karst , Age-dependent shift in spontaneous excitation-inhibition balance of infralimbic prefrontal layer II/III neurons is accelerated by early life stress, independent of forebrain mineralocorticoid receptor expression. Neuropharmacology **180**, 108294 (2020). 32882227 10.1016/j.neuropharm.2020.108294

[56] M. Mayford , Control of memory formation through regulated expression of a CaMKII transgene. Science **274**, 1678–1683 (1996). 8939850 10.1126/science.274.5293.1678

[57] D. S. Sun , Repeated restraint stress led to cognitive dysfunction by NMDA receptor-mediated hippocampal CA3 dendritic spine impairments in Juvenile Sprague-Dawley rats. Front. Mol. Neurosci. **13**, 552787 (2020). 33192290 10.3389/fnmol.2020.552787 PMC7604534

[58] A. M. Magarinos, B. S. McEwen, Stress-induced atrophy of apical dendrites of hippocampal CA3c neurons: Involvement of glucocorticoid secretion and excitatory amino acid receptors. Neuroscience **69**, 89–98 (1995). 8637636 10.1016/0306-4522(95)00259-l

[59] N. Li , Glutamate N-methyl-D-aspartate receptor antagonists rapidly reverse behavioral and synaptic deficits caused by chronic stress exposure. Biol. Psychiatry **69**, 754–761 (2011). 21292242 10.1016/j.biopsych.2010.12.015 PMC3068225

[60] J. L. McGaugh, Memory–a century of consolidation. Science **287**, 248–251 (2000). 10634773 10.1126/science.287.5451.248

[61] D. Norris, Short-term memory and long-term memory are still different. Psychol. Bull. **143**, 992–1009 (2017). 28530428 10.1037/bul0000108 PMC5578362

[62] P. J. Barnes, How corticosteroids control inflammation: Quintiles prize lecture 2005. Br. J. Pharmacol. **148**, 245–254 (2006). 16604091 10.1038/sj.bjp.0706736 PMC1751559

[63] A. E. Coutinho, K. E. Chapman, The anti-inflammatory and immunosuppressive effects of glucocorticoids, recent developments and mechanistic insights. Mol. Cell. Endocrinol. **335**, 2–13 (2011). 20398732 10.1016/j.mce.2010.04.005 PMC3047790

[64] J. S. Takahashi, Transcriptional architecture of the mammalian circadian clock. Nat. Rev. Genet. **18**, 164–179 (2016). 27990019 10.1038/nrg.2016.150 PMC5501165

[65] K. Begemann, A. M. Neumann, H. Oster, Regulation and function of extra-SCN circadian oscillators in the brain. Acta Physiol. (Oxf.) **229**, e13446 (2020). 31965726 10.1111/apha.13446

[66] L. E. Chun , Adrenal-dependent and -independent stress-induced Per1 mRNA in hypothalamic paraventricular nucleus and prefrontal cortex of male and female rats. Stress **21**, 69–83 (2018). 29165002 10.1080/10253890.2017.1404571

[67] B. P. Flynn , Corticosterone pattern-dependent glucocorticoid receptor binding and transcriptional regulation within the liver. PLOS Genet. **17**, e1009737 (2021). 34375333 10.1371/journal.pgen.1009737 PMC8378686

[68] M. Joëls, Corticosteroids and the brain. J. Endocrinol. **238**, R121–R130 (2018). 29875162 10.1530/JOE-18-0226

[69] A. Balsalobre , Resetting of circadian time in peripheral tissues by glucocorticoid signaling. Science. **289**, 2344–2347 (2000). 11009419 10.1126/science.289.5488.2344

[70] M. Joëls, Corticosteroid actions in the hippocampus. J. Neuroendocrinol. **13**, 657–669 (2001). 11489082 10.1046/j.1365-2826.2001.00688.x

[71] V. R. Soares , Peripheral clock system circadian abnormalities in Cushing’s disease. Chronobiol. Int. **37**, 867–876 (2020). 32354240 10.1080/07420528.2020.1758126

[72] K. D. Young, S. H. Preskorn, Neuroscientific basis of corticosteroid-induced changes in human cognitive and emotional processing. J. Psychiatr. Pract. **19**, 309–315 (2013). 23852106 10.1097/01.pra.0000432601.09514.12

[73] C. M. Pariante, A. H. Miller, Glucocorticoid receptors in major depression: Relevance to pathophysiology and treatment. Biol. Psychiatry **49**, 391–404 (2001). 11274650 10.1016/s0006-3223(00)01088-x

[74] M. T. Birnie , Time of day influences stress hormone response to ketamine. J. Neuroendocrinol. **34**, e13194 (2022). 36056546 10.1111/jne.13194 PMC9787621

[75] T. V. P. Bliss, G. L. Collingridge, A synaptic model of memory: Long-term potentiation in the hippocampus. Nature **361**, 31–39 (1993). 8421494 10.1038/361031a0

[76] S. Nabavi , Engineering a memory with LTD and LTP. Nature **511**, 348–352 (2014). 24896183 10.1038/nature13294 PMC4210354

[77] M. Yasuda, M. R. Mayford, CaMKII Activation in the entorhinal cortex disrupts previously encoded spatial memory. Neuron **50**, 309–318 (2006). 16630840 10.1016/j.neuron.2006.03.035

[78] W. Plihal, R. Pietrowsky, J. Born, Dexamethasone blocks sleep induced improvement of declarative memory. Psychoneuroendocrinology **24**, 313–331 (1999). 10101736 10.1016/s0306-4530(98)00080-8

[79] A. Rashidy-Pour, H. Sadeghi, A. A. Taherain, A. A. Vafaei, Y. Fathollahi, The effects of acute restraint stress and dexamethasone on retrieval of long-term memory in rats: An interaction with opiate system. Behav. Brain Res. **154**, 193–198 (2004). 15302125 10.1016/j.bbr.2004.02.007

[80] A. Barsegyan, S. M. Mackenzie, B. D. Kurose, J. L. McGaugh, B. Roozendaal, Glucocorticoids in the prefrontal cortex enhance memory consolidation and impair working memory by a common neural mechanism. Proc. Natl. Acad. Sci. U.S.A. **107**, 16655–16660 (2010). 20810923 10.1073/pnas.1011975107 PMC2944727

[81] D. de Quervain, L. Schwabe, B. Roozendaal, Stress, glucocorticoids and memory: Implications for treating fear-related disorders. Nat. Rev. Neurosci. **18**, 7–19 (2017). 27881856 10.1038/nrn.2016.155

[82] R. Vierk , Aromatase inhibition abolishes LTP generation in female but not in male mice. J. Neurosci. **32**, 8116–8126 (2012). 22699893 10.1523/JNEUROSCI.5319-11.2012 PMC6703647

[83] W. Wang , Memory-related synaptic plasticity is sexually dimorphic in rodent hippocampus. J. Neurosci. **38**, 7935–7951 (2018). 30209204 10.1523/JNEUROSCI.0801-18.2018 PMC6136152

[84] R. A. McKinney, M. Capogna, R. Dürr, B. H. Gähwiler, S. M. Thompson, Miniature synaptic events maintain dendritic spines via AMPA receptor activation. Nat. Neurosci. **2**, 44–49 (1999). 10195179 10.1038/4548

[85] B. Gibbison , Corticosteroids in septic shock: A systematic review and network meta-analysis. Crit. Care **21**, 1–8 (2017). 28351429 10.1186/s13054-017-1659-4 PMC5371269

[86] Y. Wang , A retrospective cohort study of methylprednisolone therapy in severe patients with COVID-19 pneumonia. Signal Transduct. Target. Ther. **5**, 1–3 (2020). 32341331 10.1038/s41392-020-0158-2 PMC7186116

[87] H. C. Atkinson, B. J. Waddell, Circadian variation in basal plasma corticosterone and adrenocorticotropin in the rat: Sexual dimorphism and changes across the estrous cycle. Endocrinology **138**, 3842–3848 (1997). 9275073 10.1210/endo.138.9.5395

[88] Y. Tahara , Entrainment of the mouse circadian clock by sub-acute physical and psychological stress. Sci. Rep. **5**, 11417 (2015). 26073568 10.1038/srep11417 PMC4466793

[89] G. Paxinos, C. R. Watson, The Rat Brain in Stereotaxic Coordinates (Academic Press, San Diego, ed. 2, 2007).

[90] M. Yoshimura , The gene expression of the hypothalamic feeding-regulating peptides incisplatin-induced anorexic rats. Peptides **46**, 13–19 (2013). 23684922 10.1016/j.peptides.2013.04.019

[91] B. L. Conway-Campbell , Glucocorticoid ultradian rhythmicity directs cyclical gene pulsing of the clock gene period 1 in rat hippocampus. J. Neuroendocrinol. **22**, 1093–1100 (2010). 20649850 10.1111/j.1365-2826.2010.02051.x PMC4968637

[92] E. Afgan , The Galaxy platform for accessible, reproducible and collaborative biomedical analyses: 2018 update. Nucleic Acids Res. **46**, W537–W544 (2018). 29790989 10.1093/nar/gky379 PMC6030816

[93] C. Trapnell , Differential gene and transcript expression analysis of RNA-seq experiments with TopHat and Cufflinks. Nat. Protoc. **7**, 562–578 (2012). 22383036 10.1038/nprot.2012.016 PMC3334321

[94] Y. Sha, J. H. Phan, M. D. Wang, “Effect of low-expression gene filtering on detection of differentially expressed genes in RNA-seq data” in 2015 37th Annual International Conference of the IEEE Engineering in Medicine and Biology Society (EMBC) (IEEE, 2015), pp. 6461–6464, 10.1109/EMBC.2015.7319872. PMC4983442 26737772

[95] A. Conesa , A survey of best practices for RNA-seq data analysis. Genome Biol. **17**, 13 (2016). 26813401 10.1186/s13059-016-0881-8 PMC4728800

[96] D. W. Huang, B. T. Sherman, R. A. Lempicki, Systematic and integrative analysis of large gene lists using DAVID bioinformatics resources. Nat. Protoc. **4**, 44–57 (2009). 19131956 10.1038/nprot.2008.211

[97] W. W. Anderson, G. L. Collingridge, Capabilities of the WinLTP data acquisition program extending beyond basic LTP experimental functions. J. Neurosci. Methods **162**, 346–356 (2007). 17306885 10.1016/j.jneumeth.2006.12.018

[98] M. T. Birnie, S. L. Lightman, B. L. Conway-Campbell, Genome-wide RNAseq from multi time point (every 4 hours) rat hippocampi with vehicle and methylprednisolone (MPL) treatment. Gene Expression Omnibus. https://www.ncbi.nlm.nih.gov/geo/query/acc.cgi?acc=GSE223761. Deposited 26 January 2023.

[99] M. T. Birnie, S. L. Lightman, B. L. Conway-Campbell. Genome-wide RNAseq of hippocampi from single time point (2 hours post 0.75 mg/mL corticosterone injection) in adrenalectomized rats. Gene Expression Omnibus. https://www.ncbi.nlm.nih.gov/geo/query/acc.cgi?acc=GSE224038. Deposited 30 Jan 2023.

