# Supplementary material for: Circadian regulation of hippocampal function is disrupted with corticosteroid treatment
Source: Proc Natl Acad Sci U S A. 2023 Apr 6;120(15):e2211996120. doi: 10.1073/pnas.2211996120 (PMC10104554; doi:10.1073/pnas.2211996120)
Supplement: Supplementary file 1 — Appendix 01 (PDF) [file pnas.2211996120.sapp.pdf]

Supporting Information for

Circadian regulation of hippocampal function is disrupted with corticosteroid treatment

Matthew T. Birnie[1†], Matthew D.B. Claydon[1,2†], Oliver Troy[1], Benjamin P. Flynn[1], Mitsuhiro Yoshimura[1], Yvonne M. Kershaw[1], Zidong Zhao[1], Rebecca C.R. Demski-Allen[1,2], Gareth R.I. Barker[2], E. Clea Warburton[2], Zuner A. Bortolotto[2], Stafford L. Lightman[1], Becky L. Conway-Campbell[1]

Matthew T. Birnie
Email: mbirnie@uci.edu

This PDF file includes:

Figures S1 to S4

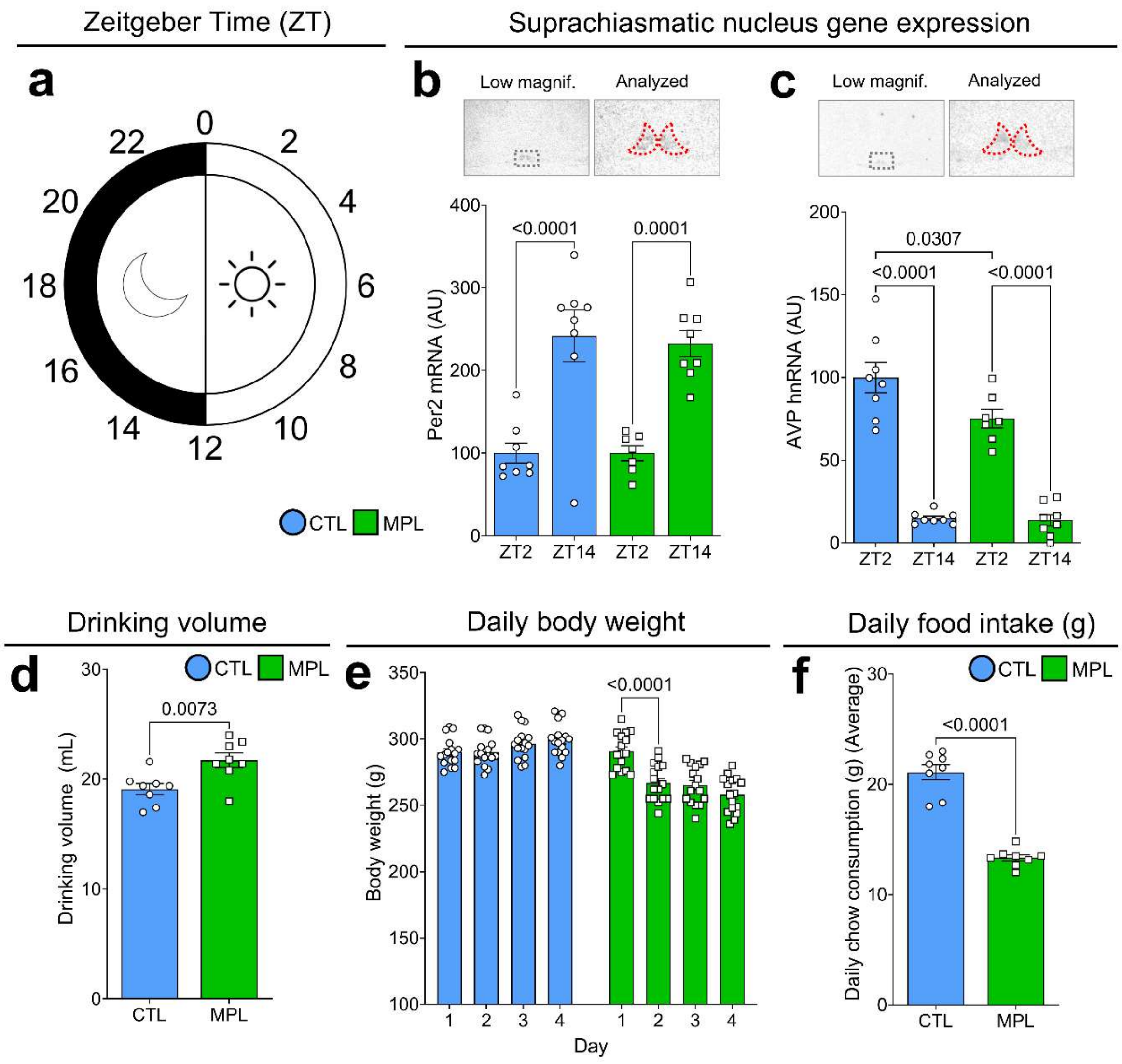

Fig. S1. Effect of methylprednisolone treatment on clock gene expression in the SCN and metabolism.

(**a**) Schematic showing zeitgeber time relative to light/dark cycle. (**b**) Period 2 mRNA expression in the SCN of CTL and MPL treated rats. $F_{1,27} = 48.41$, $P < 0.0001$, for time by two-way ANOVA. ****$P < 0.0001$, ***$P = 0.0001$, Sidak's multiple comparisons test. (**c**) AVP hnRNA expression in the SCN of CTL and MPL treated rats. $F_{1,27} = 166.1$, $P < 0.0001$, for time by two-way ANOVA. $F_{1,27} = 5.283$, $P = 0.0295$, for treatment by two-way ANOVA. $F_{1,27} = 4.316$, $P = 0.0474$, for an interaction. ****$P < 0.0001$, *$P = 0.0307$. Sidak's multiple comparisons test. (**d**) Average drinking volume across treatment period. $P = 0.0073$, unpaired *t*-test. (**e**) Body weight measured across the treatment period shows MPL treated rats lost weight on the initial day, but stabilized after. $F_{3,120} = 6.517$, $P = 0.0004$, for day by two-way ANOVA. $P < 0.0001$, Sidak's multiple comparison. (**f**) MPL treatment suppressed regular chow consumption. $P < 0.0001$, unpaired *t*-test. Data are mean ± s.e.m. *P<0.05.

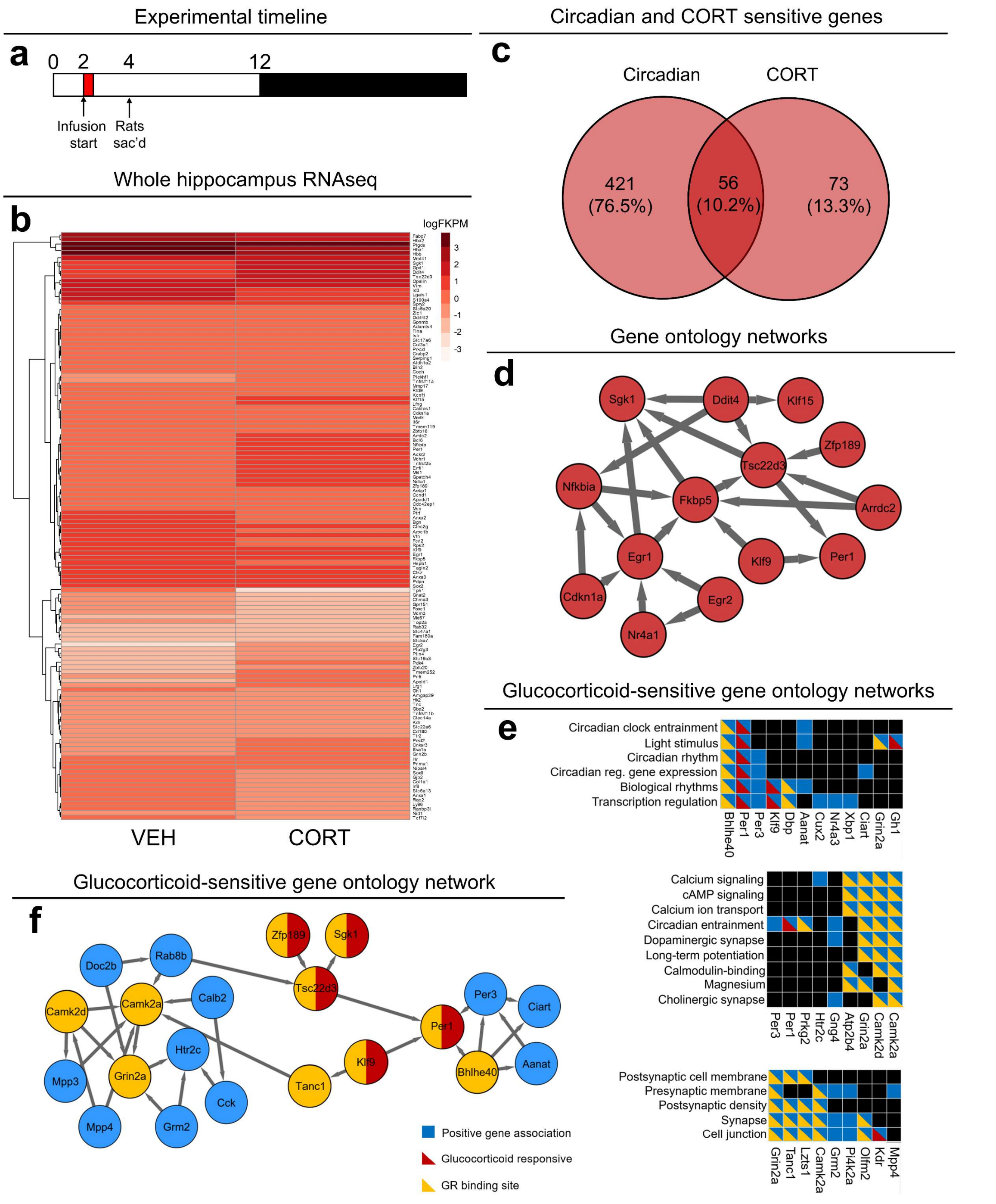

Fig. S2. Hippocampal RNAseq of Vehicle and CORT infused ADX rats.
(**a**) Schematic of timing of VEH or CORT infusion and hippocampi collection. (**b**) Heatmap of DEGs from vehicle infused and CORT infused ADX rats (n = 4 vehicle, n = 4 CORT). (**c**) Of total DEGs in Fig. 1, 56 (10.2%) DEGs are regulated by glucocorticoids. (**d**) Representation of a regulated signaling network enrichment analysis of glucocorticoid responsive genes that are under circadian control. (**e**) Overlap of functional clustering of DEGs at ZT 10 from Fig. 1, with identified glucocorticoid responsive (red) and associated GR binding site (yellow). (**f**) Representation of a regulated signaling network enrichment analysis genes that are under circadian control (blue), glucocorticoid responsive (red), and/or contain a GR binding site (yellow).

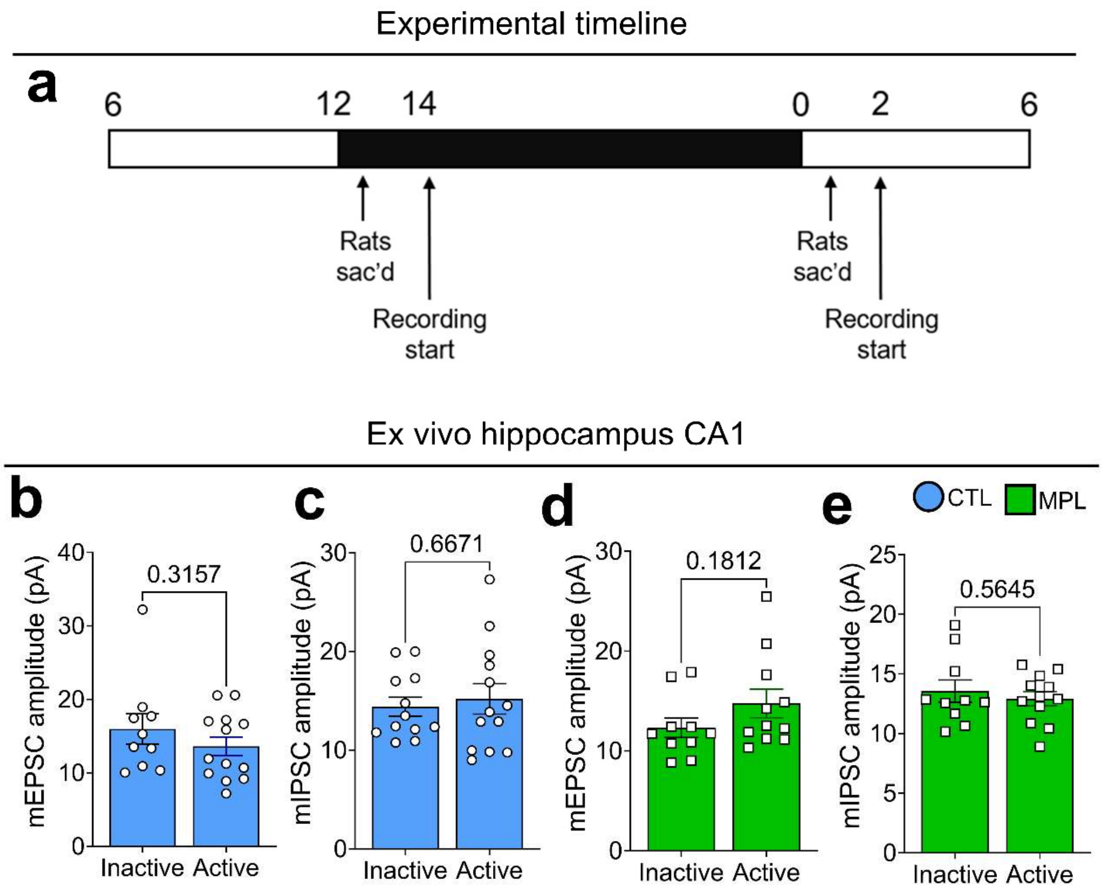

Fig. S3. mEPSC or mIPSC amplitudes were not influenced by time of day or methylprednisolone-treatment.

(**a**) Schematic of timing of electrophysiology experiments. (**b**) Time of day did not affect the amplitude of mEPSCs (n = 10 inactive; n = 12 active, $P = 0.3157$) nor (**c**) mIPSCs (n = 12 inactive; n = 13 active, $P = 0.6671$). MPL treatment did not influence time of day (**d**) mEPSCs (n = 10 inactive; n = 11 active, $P = 0.1812$) or (**e**) mIPSCs (n = 10 inactive; n = 12 active, $P = 0.5645$).

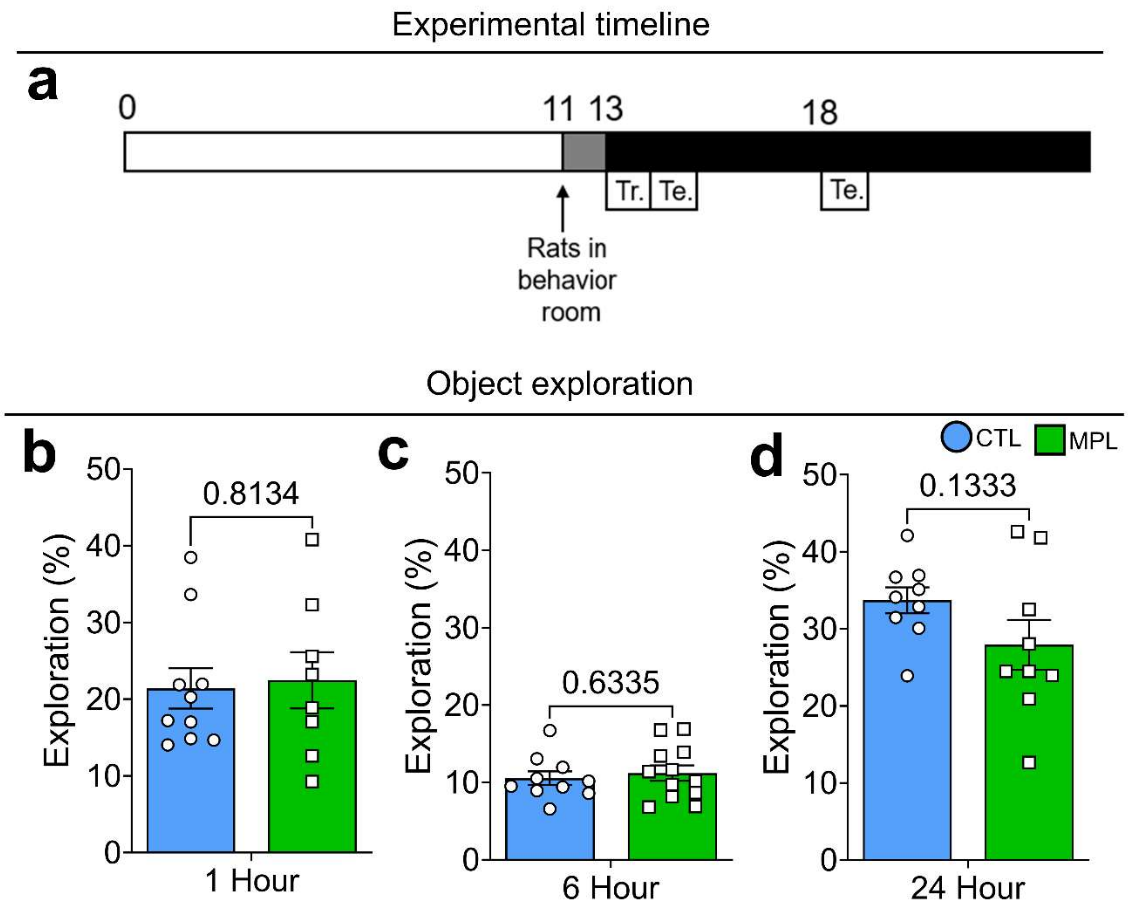

Fig. S4. Methylprednisolone did not influence exploration during object location memory testing.

(**a**) Schematic of object location experimental timing. (**b**) Exploration of objects in CTL and MPL treated rats 1hr post sample phase (n = 10 CTL; 8 MPL, P = 0.8134) (**c**) Object exploration in CTL and MPL treated rats 6hr post sample phase (n = 10 CTL; 12 MPL, P = 0.6335). (**d**) Object exploration in CTL and MPL treated rats 24hr post sample phase (n = 9 CTL; 9 MPL, P = 0.1333), Data are mean ± s.e.m. Tr; Training. Te; Test.
